# Strain Engineered Band Gaps and Electronic Properties in PbPdO_2_ and PbPd_0.75_Co_0.25_O_2_ Slabs

**DOI:** 10.3390/ma11102002

**Published:** 2018-10-16

**Authors:** Yanmin Yang, Kehua Zhong, Guigui Xu, Jian-Min Zhang, Zhigao Huang

**Affiliations:** 1 Fujian Provincial Key Laboratory of Quantum Manipulation and New Energy Materials, College of Physics and Energy, Fujian Normal University, Fuzhou 350117, China; yym@fjnu.edu.cn (Y.Y.); khzhong@fjnu.edu.cn (K.Z.); 2 Fujian Provincial Collaborative Innovation Center for Optoelectronic Semiconductors and Efficient Devices, Xiamen 361005, China; 3 Concord University College, Fujian Normal University, Fuzhou 350117, China; xuguigui082@126.com

**Keywords:** PbPdO_2_, strain, band gap, piezoresistance, anisotropic, first-principles calculations

## Abstract

Electronic structure and corresponding electrical properties of PbPdO_2_ and PbPd_0.75_Co_0.25_O_2_ ultrathin slabs with (002) preferred orientation were systematically investigated using first-principles calculations. The calculated results revealed the strain induced evidently the changes of band structure and carrier concentration in both slabs. It was also found that PbPdO_2_ and PbPd_0.75_Co_0.25_O_2_ ultrathin slabs exhibited evident differences in the external strain dependence of the band gap and charge carrier concentration, which was strongly dependent on bond angle and bond length induced by in-plane anisotropy strain. Interestingly, the carrier concentration of the PbPd_0.75_Co_0.25_O_2_ slab could increase up to 5–6 orders of magnitude with the help of external strain, which could explain the potential mechanism behind the observed colossal strain-induced electrical behaviors. This work demonstrated that the influence of the doping effect in the case of PbPdO_2_ could be a potentially fruitful approach for the development of promising piezoresistive materials.

## 1. Introduction

In the past decade, spin-gapless semiconductors (SGS) have attracted increasing interest because of their unique physical properties, leading to their potential application in electronic devices, such as field-effect transistors, optoelectronics, electronic sensors, and supercapacitors, amongst others [1,2,3,4,5,6]. Among them, the PbPdO_2_-based spin gapless semiconductor is considered as a promising candidate because of its non-toxicity, compatibility to the oxide semiconductor devices, and sensitivity to the doping metal elements, electric field, and operation current. Based on local density approximation calculations, the oxide-based PbPdO_2_ gapless semiconductor was firstly discovered by Wang [4]. Following this, extensive investigations on the electric and magnetic properties of PbPdO_2_-based semiconductors were carried out theoretically and experimentally. Wang et al. studied the roles of both electrical current and magnetic field on the resistivity of PbPd_0.75_Co_0.25_O_2_ thin films, and unusual colossal electroresistance and magnetoresistance were observed [5]. Moreover, the distinct different magnetoresistance effects were observed in PbPd_0.9_Cu_0.1_O_2_ and PbPd_0.9_Zn_0.1_O_2_, which would be attributed to local structure deformation due to Pd/O deficiencies [7]. Based on the bound magnetic polaron (BMP) theory, the potential mechanism behind the observed ferromagnetic, paramagnetic, and antiferromagnetic properties coexisting in Co–doped PbPdO_2_ film were suggested [8]. It was suggested that Pd–O hybridization in Co–doped PbPdO_2_ thin films were responsible for the transition from weak localization and weak anti-localization [9]. Simultaneously, many studies on valence states and band structures have been carried out to understand the mechanism on the observed physical behaviors. For example, Chen et al. revealed that a small band gap of PbPdO_2_ would be induced by Pd deficiency in composites, resulting in increased hybridization of O(2p)–Pb(6p) and decreased O(2p)–Pd(4d) hybridizations [10]. It was found that Pd–O hybridization would efficiently mediate the magnetic coupling among Co atoms [11]. The ferromagnetism and paramagnetism were found to coexist in the Fe–doped PbPdO_2_, and the bound magnetic polaron model was used to account for the ferromagnetism origin [12]. Based on the measured electronic structures, Pb(Pd_0.9_T_0.1_)O_2_ (T = Mn, Co) oxides were found to be the small-gap semiconductors [13]. In our previous study, it was observed that the external electric field in PbPdO_2_ slab with (002)-preferred orientation influences sensitively the band gap and carrier concentration, which explains the extraordinary electrical behaviors [14]. 

Recently, the strain effects on the physical properties of two-dimensional materials were investigated [15,16,17,18]. The direct-indirect band gap transition induced by strain was also found in two-dimensional phosphorene, which is explained by the near-band-edge electronic orbital theory [15]. It has been established that the strain induces modulation of the band gap resulting in a piezoresistive effect in silicon [16]. Recently, the piezoresistive effect was also found in single-atomic-layer and atomically thin MoS_2_ films [17,18]. Strain dependent carrier concentration is generally characterized with piezoresistive gauge factor. The gauge factor (GF) of piezoresistance can be calculated as follows [18],

(1) $$\mathrm{GF}=\frac{\Delta \rho }{\rho }/\varepsilon$$

where $\rho_{0}$ is the resistance without strain and Δρ is change of resistance with strain *ε*. The resistivity (ρ) is in inverse proportion to carrier concentration (*n*). As ε = 0, let *n* = *n*_0_; ε ≠ 0, let *n* = $n_{\varepsilon }$. The gauge factor can be re-expressed as follows,

(2) $$\mathrm{GF}=\frac{\Delta \rho }{\rho }/\varepsilon \propto (\frac{n_{0}}{n_{\varepsilon }}-1)/\varepsilon$$

In PbPdO_2_-based composites, different preparation and processing methods result unavoidably in different microstructure and strain states, which consequently influences the band gap, carrier concentration, and corresponding electrical properties. Specifically, being similar to the layered MoS_2_, (002) preferred orientation layered PbPdO_2_ has a small band gap [14], and the piezoresistive effect is expected. 

In this work, based on the first-principle calculation method, a plane strain model was set up to obtain a deformed lattice with in-plane arbitrary uniaxial strain. In-plane anisotropy strain dependence of band-gap and carrier concentration were systematically investigated in the PbPdO_2_ and PbPd_0.75_Co_0.25_O_2_ slabs with preferred (002) orientation. These results can be well explained according to the p-d exchange interaction. Moreover, it is strongly suggested that the element-doping PbPdO_2_ should become an important piezoresistance candidate material.

## 2. Methods

Based on Vienna ab initio simulation package (VASP), the self-consistent total energy was calculated and the geometry was optimized using the perdew-burke-ernzerhof (PBE) exchange-correlation functionals and the projector-augmented wave potentials [19,20]. The strain effect on the electronic properties of PbPdO_2_ and PbPd_0.75_Co_0.25_O_2_ slabs were simulated via standard DFT (Density functional theory) with generalized gradient approximation (GGA) method [21].

The cut-off energy was set to be 500 eV. The initial structure of PbPdO_2_ and PbPd_0.75_Co_0.25_O_2_ slabs were obtained from bulk PbPdO_2_ and PbPd_0.75_Co_0.25_O_2_, respectively [10,20]. Then, to make the in-plane force reach up to the minimum, both initial structures were totally relaxed through the energy minimization method. Starting with the relaxed initial structures of PbPdO_2_ and PbPd_0.75_Co_0.25_O_2_ slabs, the effects on the atoms and band structures were studied systematically under the strain with the range of ±2%, which applied in the in-plane anisotropy uniaxial strain direction. The positive (negative) values of strain corresponded to the stretch and compression, respectively. The positions of all the atoms in the cell were relaxed by the optimizations of the strained structures with the Gaussians smearing method. After relaxation, each atom’s convergence tolerance of force was smaller than 0.01 eV/Å. Meanwhile, 21 × 11 × 1 and 19 × 13 × 1 Monkhorst-Pack’s meshes were used in the calculation of density of states (DOS) for PbPdO_2_ and PbPd_0.75_Co_0.25_O_2_ slabs, respectively.

## 3. Results and Discussion

**Figure 1:**
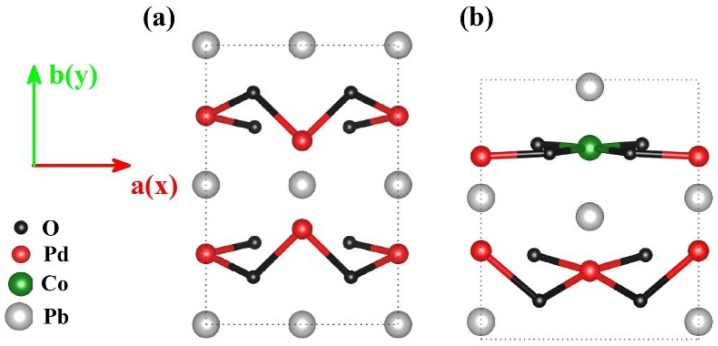
Top view of atomic structures in ab plane, (**a**) PbPdO_2_, and (**b**) PbPd_0.75_Co_0.25_O_2_ with (002) orientation.

Figure 1 shows the relaxed crystal structures of PbPdO_2_ and PbPd_0.75_Co_0.25_O_2_ ultrathin slabs with (002) preferred orientation. As shown in Figure 1, the unstrained PbPdO_2_ and ultrathin slab revealed an in-plane symmetric configuration, maintaining the important properties of PbPdO_2_. After Co-doping, the symmetric configuration of PbPd_0.75_Co_0.25_O_2_ was broken. In comparison to the pristine PbPdO_2_, the broken-symmetry in PbPd_0.75_Co_0.25_O_2_ was expected to bring different physical properties. It should be noted that the anisotropy of PbPd_0.75_Co_0.25_O_2_ was greatly affected by the state of the Co-substitution. Moreover, its configurations were fairly complicated. Here, the configuration with the least number of atoms and only one Co doping atom was considered, as seen in Figure 1b. Specifically, this Co atom forms square planar bonding with the nearest-neighbor four O atoms, which plays an important role on the electrical properties of PbPd_0.75_Co_0.25_O_2_. 

**Figure 2:**
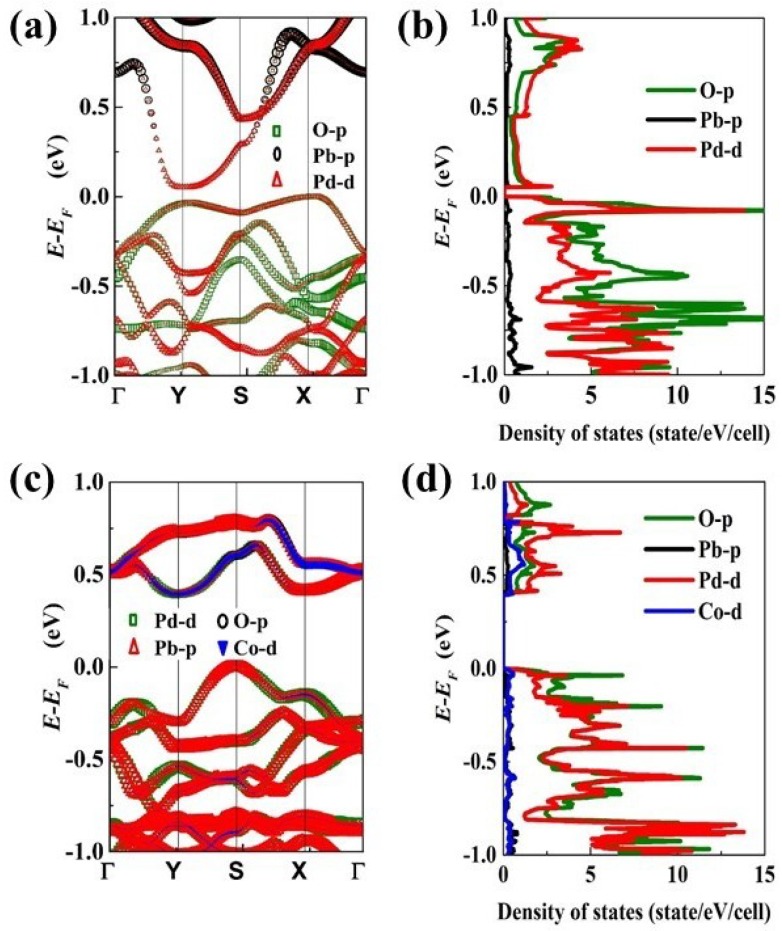
(**a**) electronic structures, and (**b**) orbital-resolved partial density of states (DOS) of (002) orientation PbPdO_2_ slab; (**c**) electronic structures, and (**d**) orbital-resolved partial DOS of (211) orientation PbPd_0.75_Co_0.25_O_2_ slab. The abscissa in (**a**,**c**) is the path of high symmetry points in Brillouin zone.

Figure 2a–d shows the calculated electronic structures and the partial densities of states of Pb, Pd, O, and Co in PbPdO_2_ and PbPd_0.75_Co_0.25_O_2_ slabs, respectively. It was found that PbPdO_2_ exhibits intrinsic characteristics of narrow band gap (0.051 eV), which was much smaller than that (0.4 eV) of the PbPd_0.75_Co_0.25_O_2_ slab. In our previous experimental work, PbPdO_2_ with (002) preferred orientation was prepared, and its band gap was found to be close to zero [22]. It is suggested that the localized Co would be responsible for the large band gap of 0.35 eV for the PbPd_0.75_Co_0.25_O_2_ bulk material. Interestingly, our calculated results were consistent with the reported results [23]. The band gap of PbPdO_2_ slab was slightly larger than that reported in our previous calculated work because of the different full in-plane structure relaxation [14]. From the density of electronic states in Figure 2b,d, it is found that DOSs of the pristine and Co-doped PbPdO_2_ slabs at minimum of the conduction band and maximum of the valence band were mainly composed of 4d(Pd) and 2p(O) states. These results were similar to those found by many research groups [6,10]. In contrast, the PbPd_0.75_Co_0.25_O_2_ slab had more distinct contribution of hybridization of 2p(O)–4d(Pd) states, where a Co 3d state added modified energy to the DOS at the Fermi energy level. 

**Figure 3:**
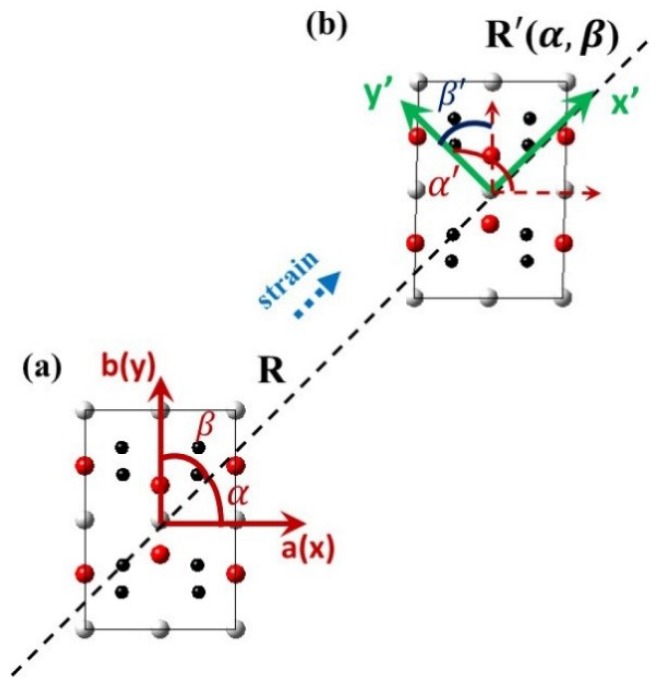
Diagram for (**a**) an undeformed lattice, (**b**) a deformed lattice with in-plane arbitrary uniaxial tensile strain (directional cosines (cosα,cosβ)).

To gain insight into the potential mechanism of strain-induced electronic properties in PbPdO_2_-base composites, a plane-stress-strain model was set up. Figure 3a,b present the undeformed lattice and a deformed lattice with in-plane arbitrary uniaxial tensile strain (directional cosines (cosα,cosβ)), respectively. The strain-related components could be obtained based on the coordinate transformation method. Assuming uniaxial strain ε is along the x′ direction in an unprimed coordinate system x-y, the strain tensor elements in the primed coordinate system are given as follows,

(2) $$\left[\begin{array}{c} \varepsilon_{x}\\ \varepsilon_{y}\\ \gamma_{xy} \end{array}\right]=\left[\begin{array}{ccc} l_{1}^{2} & l_{2}^{2} & l_{1}l_{2}\\ m_{1}^{2} & m_{2}^{2} & m_{1}m_{2}\\ 2l_{1}m_{1} & 2l_{2}m_{2} & l_{1}m_{2}+l_{2}m_{1} \end{array}\right]\left[\begin{array}{c} \varepsilon_{x^{\prime }}\\ \varepsilon_{y^{\prime }}\\ \gamma_{x^{\prime }y^{\prime }} \end{array}\right]$$

where $\varepsilon_{x^{\prime }}$($\varepsilon_{y^{\prime }}$) and $\gamma_{x^{\prime }y^{\prime }}$ are normal (tensile or compressive) and shear strains, respectively [24]. The directional cosines are

(3) $$l_{1}=\cos\alpha ,\;l_{2}=\cos\alpha^{\prime }$$

(4) $$m_{1}=\cos\beta ,\;m_{2}=\cos\beta^{\prime }$$

where α, β, $\alpha^{\prime }$, $\beta^{\prime }$ are arbitrary directions in the x-y and x′-y′ coordinate system, as seen in Figure 3.

A deformation of the unit cell is created by changing the Bravais lattice vectors *R* of the undeformed unit cell to *R*′ using a strain matrix as follows:

(5) $$R^{\prime }\left(\alpha ,\beta \right)=R\left[\begin{array}{ccc} 1+\varepsilon_{x} & \gamma_{xy} & 0\\ \gamma_{xy} & 1+\varepsilon_{y} & 0\\ 0 & 0 & 1 \end{array}\right]$$

where *R* is the Bravais lattice vectors with strain, and *R*′ is the Bravais lattice vectors without strain, $\varepsilon_{x}$($\varepsilon_{y}$) and $\gamma_{xy}$($\gamma_{yx}$) are the normal (tensile or compressive) and shear strain-related components, respectively.

**Figure 4:**
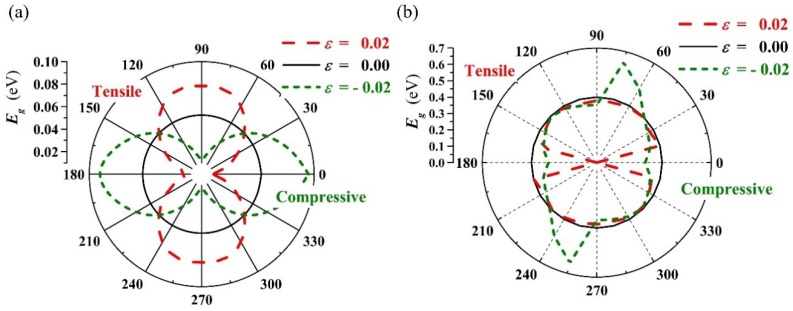
The orientation distribution curves of band gap $E_{g}$ for (**a**) PbPdO_2_, and (**b**) PbPd_0.75_Co_0.25_O_2_ with ε = −0.02, 0.00, 0.02.

Figure 4a,b shows the orientation distribution curves of band gap $E_{g}$ with ε = −0.02, 0.00, 0.02 for the PbPdO_2_ and PbPd_0.75_Co_0.25_O_2_ slabs, respectively. It is clear that both slabs show a distinct anisotropy of gap with different strain. In Figure 4a, the PbPdO_2_ slab shows the symmetrical and peanut-like $E_{g}-\alpha$ curves, and having the largest and smallest band-gap values along the x axis ($\alpha {=0}^{\circ }$) or y axis ($\alpha {=90}^{\circ }$), respectively. In contrast, as shown in Figure 4b, the PbPd_0.75_Co_0.25_O_2_ slab has a maximum gap for the strain ε = −0.02 at about $\alpha {=75}^{\circ }$ and a minimum gap for the strain ε = 0.02 at about $\alpha {=0}^{\circ }$. 

**Figure 5:**
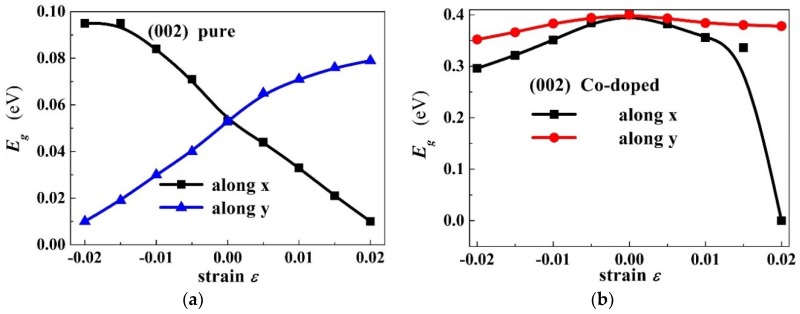
Band gap $E_{g}$ as a function of strain for (**a**) PbPdO_2_, and (**b**) PbPd_0.75_Co_0.25_O_2_ along x-axis (called x) and b-axis (called y).

Figure 5a,b shows the band gap as a function of the strain for PbPdO_2_ and PbPd_0.75_Co_0.25_O_2_ along x axis ($\alpha {=0}^{\circ }$) and y axis ($\alpha {=90}^{\circ }$), respectively. As shown in Figure 5a, it is found that the band-gap value increases with increasing strain along the y-axis, whilst the band-gap value decreases with increasing strain along the x-axis. It is interesting that the band gap of the PbPdO_2_ slab would widen when a compressive stress is applied closely to the x-axis or a tensile stress is applied closely to the y-axis, as seen in Figure 4a. These calculated results can be explained according to the interaction of Pd–O bonding. It is expected that a compressive stress along the x-axis or tensile stress along the y-axis pulls O atoms apart from Pd atoms, which weakens the interaction of Pd and O. On the other hand, a tensile stress along the x-axis or compressive stress along the y-axis would push O atoms closely to Pd atoms and strengthen the interaction of Pd and O. As a result, the band gap is decreased. A similar result has also been reported in MoS_2_ and black phosphorus [25,26].

**Figure 6:**
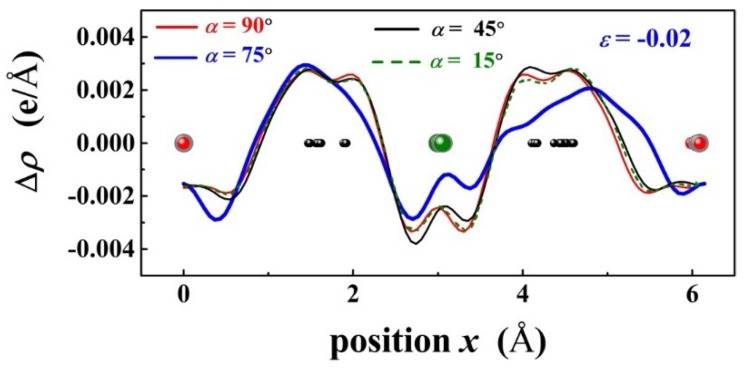
The plane averaged electron density difference Δρ of PbPd_0.75_Co_0.25_O_2_ projected along the x axis and under ε = −0.02, with *α* = 15°, 45°, 75°, and 90°, respectively.

**Figure 7:**
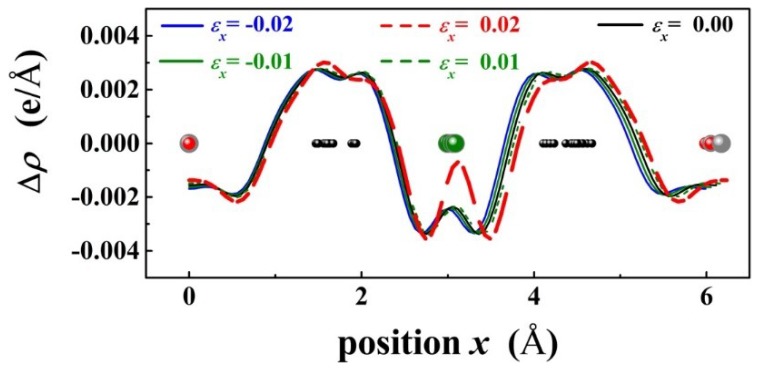
The plane averaged electron density difference Δρ of PbPd_0.75_Co_0.25_O_2_ under $\varepsilon_{x}$ = −0.02, −0.01, 0.00, 0.01, 0.02, where the projection of Δρ was along the x axis.

Figure 6 shows the plane averaged electron density difference Δ*ρ* of PbPd_0.75_Co_0.25_O_2_ projected along the x axis and under ε = −0.02, with *α* = 15°, 45°, 75°, and 90°, respectively. In comparison, it is found that p-d charge transfer Δ*ρ* between Co and O exhibits an evident change for *α* = 75°. Therefore, the band gap of PbPd_0.75_Co_0.25_O_2_ is expected to be changed significantly as the compressive stress direction is along a particular direction with *α* = 75°. Figure 7 shows the plane averaged electron density difference Δρ of PbPd_0.75_Co_0.25_O_2_ projected along the x axis under $\varepsilon_{x}$ = −0.02, −0.01, 0.00, 0.01, 0.02, respectively. Based on spin-splitting theory, the minimum gap of the PbPd_0.75_Co_0.25_O_2_ slab with the strain $\varepsilon_{x}$ = 0.02 is strongly related to the variation of Δρ. From Figure 7, it is concluded that Co atom should act as the source of the localized magnetic moment, and the coupling between the p-state from O and d-state from Co could induce a strong exchange interaction (named as p-d exchange interaction) in PbPd_0.75_Co_0.25_O_2_. Moreover, the p-d exchange interaction was found to be nearly inversely proportional to the unit cell volume [27]. Therefore, p-d exchange interaction mediated by strain should be responsible for variation of the plane averaged electron density difference, leading to a clear change of the band gap. 

**Figure 8:**
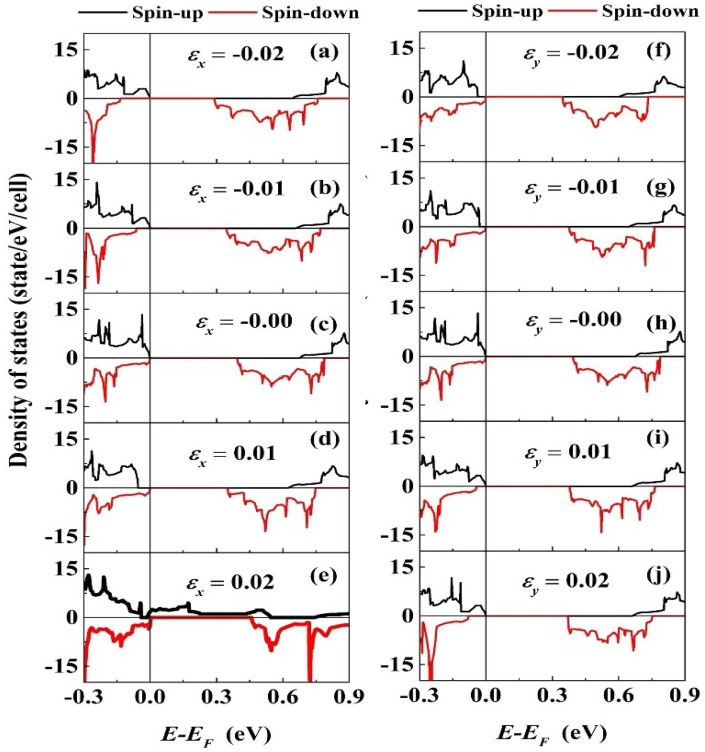
The spin polarized total density states of the PbPd_0.75_Co_0.25_O_2_ slab with different strain $\varepsilon_{x}(\varepsilon_{y})$: (**a**–**e**) along the x-axis, and (**f**–**j**) along the y-axis external uniaxial strain directions.

Figure 8 shows the spin polarized total density states of the PbPd_0.75_Co_0.25_O_2_ slab with different strain along the x and y-axes external uniaxial strain directions. From the figure, it is found that the spin-up and spin-down DOSs are asymmetric, which means the existence of magnetic moment. The magnetic properties in element doped PbPdO_2_ will be studied in subsequent research work. Interestingly, as shown in Figure 8e, the tensile strain with $\varepsilon_{x}$ = 0.02 leads to the zero-band gap structure. The strain gives rise to the evident left shift of the bottom of the conduction band in spin-down DOSs, which effectively modulates the band gap. When the strain is large enough, the top of the valence band and the bottom of the conduction band in spin-up and spin-down DOSs all shift with changing strain, which leads to clear modulation of the band gap. This interesting phenomenon is similar to the fact that the slight tensile strain results in the zero-band gap structure [4].

Unique electrical properties are highly desirable for practical application, and charge carrier concentration is a key parameter for the intrinsic semiconductor. For the intrinsic semiconductor, the charge charier concentration can be estimated as follows [28],

(6) $$n\propto T^{3/2}\exp\left[-\frac{E_{g}}{2K_{B}T}\right]$$

(7) $$n_{\varepsilon }/n_{0}\propto \exp\left[-\frac{\Delta E_{g}}{2K_{B}T}\right]=\exp\left[-\frac{E_{g}-E_{g_{0}}}{2K_{B}T}\right]$$

where *K_B_*, *E_g_* are the Boltzmann constant and band gap, respectively. In this paper, all the temperatures in carrier concentration were calculated at *T* = 100 K. As ε = 0, let *n* = *n*_0_; ε ≠ 0, let *n* = $n_{\varepsilon }$.

**Figure 9:**
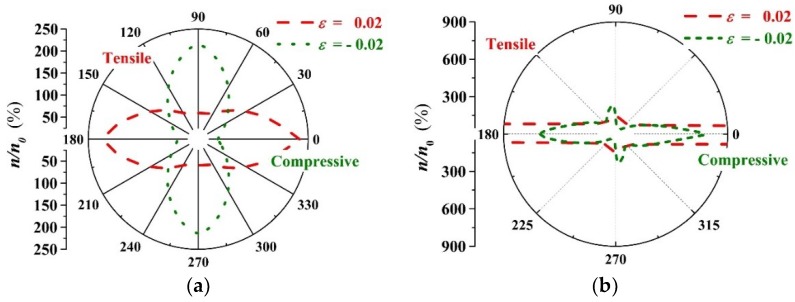
The orientation distribution curves of intrinsic charge carrier concentration ratio $n/n_{0}$ for (**a**) PbPdO_2_, and (**b**) PbPd_0.75_Co_0.25_O_2_ with ε = −0.02 and 0.02.

**Figure 10:**
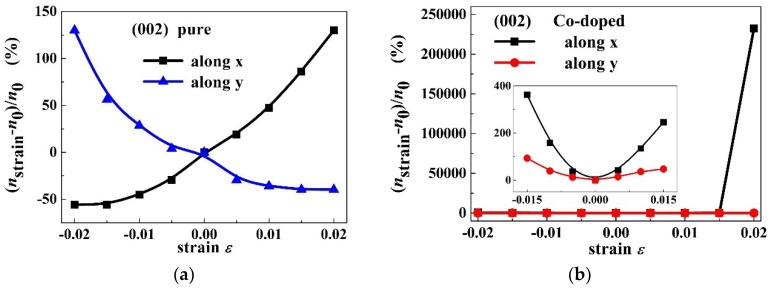
The intrinsic charge carrier concentration ratio $(n_{\varepsilon }-n_{0})/n_{0}$ as a function of strain for (**a**) PbPdO_2_, and (**b**) PbPd_0.75_Co_0.25_O_2_ along the a-axis (called x) and b-axis (called y) with ε = −0.02 and 0.02. The inset shows a magnification curve with strain ranges from −0.015 to 0.015.

Combined with the results presented in Figure 4a,b, the external strain dependence of charge carrier concentration ratio ($n_{\varepsilon }/n_{0}$) for PbPdO_2_ and PbPd_0.75_Co_0.25_O_2_ slabs were evaluated, respectively. Figure 9a,b shows the orientation distribution curves of the intrinsic charge carrier concentration ratio $n/n_{0}$ for PbPdO_2_ and PbPd_0.75_Co_0.25_O_2_ slabs with ε = −0.02, −0.01, 0.00, 0.01, 0.02, respectively. Similar to the dependence of band gap $E_{g}$ on strain orientation, PbPd_0.75_Co_0.25_O_2_ exhibits more distinct anisotropy in carrier concentration with strain direction, especially along the x-axis. As shown in Figure 9a, the pristine PbPdO_2_ slab demonstrates the symmetrical and olive-like ${\left(n_{\varepsilon }/n_{0}\right)}_{-\alpha }$ curves, and having its largest and smallest band-gap values along the x-axis or y-axis, respectively. For the PbPd_0.75_Co_0.25_O_2_ slab, the carrier concentration is sensitive to the application direction of strain. When the compressive stress applies along a direction of 75° ($\alpha \approx 75^{\circ }$) and x-axis, the remarkable variety in carrier concentration appears, as shown in Figure 9b. Figure 10c,d shows the intrinsic charge carrier concentration ratio $(n_{\varepsilon }-n_{0})/n_{0}$ as a function of strain for PbPdO_2_ and PbPd_0.75_Co_0.25_O_2_ slabs along the x and y-axes, respectively. For the PbPdO_2_ slab, the carrier concentration increases monotonically with increasing compressive stress, but decreasing with increasing tensile stress along the x-axis. On the contrary, the carrier concentration decreases with increasing compressive stress, while increasing with increasing tensile stress along the y-axis. In contrast, the carrier concentration of PbPd_0.75_Co_0.25_O_2_ slab increases with increasing compressive and tensile stresses along both the x and y-axes. As the compressive stress increases beyond 0.015 along the x-axis, carrier concentration of the PbPd_0.75_Co_0.25_O_2_ slab increases rapidly. It was found that the carrier concentration of PbPd_0.75_Co_0.25_O_2_ could sharply increase up to 5–6 orders of magnitude with the help of external strain with ε = 0.02. The calculated results suggest strongly that the element-doping PbPdO_2_ should become an important piezoresistance candidate material. 

**Figure 11:**
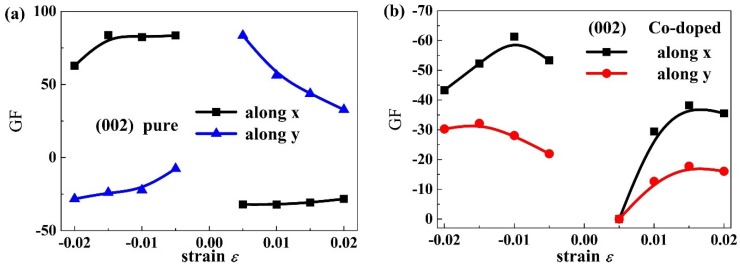
The piezoresistive gauge factor as a function of strain for (**a**) PbPdO_2_, and (**b**) PbPd_0.75_Co_0.25_O_2_ along the x and y axes.

Figure 11 presents the strain dependence of gauge factor for the PbPdO_2_ and PbPd_0.75_Co_0.25_O_2_ slab along the x and y axes. Table 1 gives some typical gauge factor values. When the tensile strain is 0.02, the piezoresistive gauge factors for the PbPdO_2_ and PbPd_0.75_Co_0.25_O_2_ slab along the x-axis are calculated to be respectively 62.8 and −43.3, which is comparable to trilayer MoS_2_ and much higher than suspended-graphene-based strain sensor [29]. As Pd–O (Co–O) polar covalent bond is different from C–C bond, PbPdO_2_-based semiconductors can exhibit a higher piezoresistive gauge factor than graphene-based strain sensors. The predicted large gauge factors in our work implies that the element-doping PbPdO_2_ may have promising opportunities to be used as strain sensors.

## 4. Conclusions

Based on first-principles calculations, the electronic structures and electrical properties of PbPdO_2_ and PbPd_0.75_Co_0.25_O_2_ ultrathin slabs were systematically investigated. The calculated results indicated that the strain induces changes of band structure and carrier concentration in both slabs. Specifically, the carrier concentration of the PbPd_0.75_Co_0.25_O_2_ slab could be modulated with 5–6 orders externally induced strain, which renders the Co-doped pristine PbPdO_2_ phase a potentially promising piezoresistive material. Moreover, the above evident external strain modulation of the band gap and carrier concentration can be well explained by spin-splitting theory.

## Figures and Tables

**Table 1:** Some typical gauge factors of PbPdO_2_ and PbPd_0.75_Co_0.25_O_2_ along the x and y axes.

Typical Gauge Factors	PbPdO_2_ Slab				PbPd_0.75_Co_0.25_O_2_ Slab			
	Direction							
	x		y		x		y	
*ε*	−0.02	0.02	−0.02	0.02	−0.02	0.02	−0.02	0.02
GF	62.8	−28.3	−28.3	32.8	−43.3	−35.5	−30.3	−16.1
